# The Story of the Insane from Year to Year

**Published:** 1892-05-07

**Authors:** 


### The Story of the Insane from Year to Year.

(Fourth Series.)

### BARNWOOD HOUSE HOSPITAL FOR THE INSANE, GLOUCESTER.

The number of the patients in this asylum fell during the year from 155 to 151. There were 25 admissions, 17 recoveries, and 4 deaths. The recoveries stand at 68 per cent., and the deaths at 2·6 per cent., both calculations being made in the way usual in asylums. Hereditary tendency accounts for 16 of the admissions, and drink for 4. Dr. Needham gives a long and valuable criticism on the new Lunacy Act, from which we extract the following: "Although the Act generally may have satisfied a sentimental outcry for a change in the law of lunacy, it has, in my opinion, made no addition to the security of the subject, as it has certainly contributed nothing towards the well-being of the patient. . . . Fortunately, there are numerous weak places in the Act, or it would, I believe, have been quite unworkable in practice. The unjust suspicions implied in some of its provisions, the want of appreciation of peculiarities of the insane condition, and the practical assumption that this legislation is not to secure the proper treatment of a disease, but the fair trial of persons accused of crimes, are, I think, all of them, unfortunate concomitants of the Act. Moreover, the statements, reports, and returns demanded by it are irritatingly and unnecessarily complicated in their forms and requirements. Under the old law it was possible for the officials of an asylum to be on the best terms with the majority of the patients, who were willing to regard them as persons who were discharging an unpleasant duty in as friendly a manner as possible. Under the new law, strictly carried out, patients must be angelic indeed if they can see in those who have charge of them other than opponents of all their best interests." Since the above was written Dr. Needham has been appointed Commissioner in Lunacy. His words will therefore carry still greater weight, and it is to be hoped they will sink into the brains of those men in high places who were responsible for presenting such an Act to the Houses of Parliament.

### LANCASHIRE ASYLUM, WHITTINGHAM.

A jump in the numbers from 1810 to 1877 was made during the year 1890. Not fewer than 396 cases were admitted; 105 were discharged recovered; and 159 died. Calculated in the usual way, the recoveries stand at 26·51 per cent., and the deaths at 8·62. Intemperance in drink is the assigned cause in 70 of the admissions, and hereditary influence is credited with being the cause in 99. Of the deaths, 61 were caused by brain disease, and 30 by consumption. We are pleased to notice Dr. Wallis's remarks about suicidal patients, and it is much to be desired that his views should become common in asylums. The real question is, does the increased freedom mean increased risk, and if so, does it also mean greater chance of recovery in the majority? Dr. Wallis believes it does, and we agree with him. The new Lunacy Act is thus spoken of: "It has involved a good deal of extra correspondence with the Commissioners, an extraordinary amount of red tapeism, and a large increase of work to the Medical Superintendents of County Asylums. The Lunacy Commissioners seem to think that the re-certification of patients should be done, as far as possible, by the Medical Superintendents. . . I cannot but regard the new Act as in every sense a mistake, and I apprehend that its operation will prove so unsatisfactory that it will bring about its own repeal or require its modification at an early date." Certainly this is a consummation devoutly to be wished for, and we are told that "good may be the final goal of ill." But we are also told that

> Faith, fanatic faith, once wedded fast
> To some vain falsehood, hugs it to the last."

It will be interesting to see which poetical quotation comes nearest the truth in this case. It would seem to be likely that another block will be added to this already far too large building. It is suggested that accommodation for 200 recent cases should be built—a block wherein the various forms of insanity could be scientifically studied. This is all very well, but Dr. Wallis is grievously mistaken if he thinks he can thereby add to his recoveries.

